# Promoting Breastfeeding in Child Care Through State Regulation

**DOI:** 10.1007/s10995-014-1560-6

**Published:** 2014-07-08

**Authors:** S. E. Benjamin Neelon, D. T. Duncan, T. Burgoine, M. Mayhew, A. Platt

**Affiliations:** 1 Department of Community and Family Medicine, Duke University Medical Center and Duke Global Health Institute, 2200 W Main Street, DUMC 104006, Durham, NC 27705 USA; 2 Duke Global Health Institute, Trent Hall, 310 Trent Drive, Durham, NC 27710 USA; 3 UKCRC Centre for Diet and Activity Research (CEDAR), Institute of Public Health, University of Cambridge, Box 296, Forvie Site, Robinson Way, Cambridge, CB2 0SR UK; 4 Department of Population Health, New York University School of Medicine, New York, NY 10016 USA

**Keywords:** Breastfeeding, Child care, Policy, Regulation

## Abstract

Policies supporting breastfeeding vary by state, but little is known about the geographical aspects of this variation. This study describes state breastfeeding licensing and administrative regulations targeting child care settings, compares regulations with national standards, and examines the spatial patterning and clustering of these regulations throughout the United States (US). We compared regulations for child care centers (centers) and family child care homes (homes) with national standards for: (1) general breastfeeding support; (2) designated place for breastfeeding; (3) no solids before infants are four months of age; and (4) no formula for breastfed infants without parent permission. We scored state regulations as 0 = standard not addressed, 1 = standard partially addressed, and 2 = standard fully addressed. We considered each regulation individually, and also summed scores to provide an overall rating of regulations by state. We mapped regulations using geographic information systems technology, and explored overall and local spatial autocorrelation using global and local variants of Moran’s *I*. Five states had regulations for centers and two for homes that addressed all four standards. Mean regulation scores were 0.35, 0.20, 0.98, 0.74 for centers, and 0.17, 0.15, 0.79, 0.58 for homes. Local Moran’s *I* revealed that New York and Pennsylvania had substantially stronger regulations than their adjacent states, while Florida had weaker regulations than its neighbors. Overall, few states had regulations that met breastfeeding standards. We identified some patterns of spatial correlation, suggesting avenues for future research to better understand distributions of regulations across the US.

## Introduction

Breastfeeding is associated with important health benefits for both mother and child. The American Academy of Pediatrics (AAP) [1] and the World Health Organization [2] recommend exclusive breastfeeding for the first six months of life and continued breastfeeding through at least one year of age. There are a number of factors that influence breastfeeding, including child care environments, where large numbers of infants are cared for in early life [3]. In the United States (US), nearly two thirds of infants spend time in non-parental care, including child care centers and family child care homes [3]. However, child care providers often receive little guidance on infant feeding. A recent study of providers found that most reported insufficient knowledge of appropriate infant feeding practices, including breastfeeding [4].

The AAP recently put forth recommendations for feeding infants in child care. The report, entitled *Caring for Our Children: National Health and Safety Performance Standards*; *Guidelines for Early Care and Education Programs, 3* ^*rd*^ *Edition (Caring for our Children)* [5] sets standards for health promotion in child care settings, including recommendations to support breastfeeding. These standards are voluntary unless mandated through state regulation. Regulations for child care facilities are enacted separately by each state. While many states use *Caring for our Children* standards as the basis for their regulations, previous studies have found substantial variation between these standards and state regulations [6–10].

A handful of studies have examined geographic differences in regulations, with mixed results. One study found that southeastern states had more regulations governing infant feeding in child care [7]. A second study examined five breastfeeding laws and found that states in the northeast had the greatest number, whereas those in the midwest had the fewest [11]. Thus, some overall geographic patterning of state level policies was evident. The extent to which regulations vary by state is not well understood, but is of policy relevance. In the absence of federal oversight, uniform policies across states provide a consistent message that mothers are supported in their decision to breastfeed their infants. Additionally, understanding geographic differences across states may help guide national efforts to promote breastfeeding. Geographic analyses can help identify areas of the country with relatively weak breastfeeding policies and target them for intervention. The purpose of this study was threefold: (1) to review current state regulations mandating support for breastfeeding in child care; (2) to compare these regulations to national breastfeeding standards; and (3) to examine spatial patterns in breastfeeding regulations across the US.

## Methods

### Data Sources

We collected data on state regulations for licensed child care facilities in early 2012. Regulation data came from a publicly available website maintained by the National Resource Center for Health and Safety in Child Care [12] and the commercial legal database WestlawNext™. Most states defined different types of child care based on the number of children in care and the location of care. Child care centers (“centers”) typically care for greater numbers of children, have more staff members, and are located in dedicated facilities. Family child care homes (“homes”) are located primarily in the residence of the provider. This provider is often the only staff member, with enrollment limited to approximately five to eight children, depending on the state. Though some states define additional categories (e.g., infant-only centers, large family child care homes), we collapsed categories into “centers” and “homes” for the purposes of this review. Since this study consisted of a regulatory review and did not involve human subjects, this research was considered exempt by the Duke University Medical Center Institutional Review Board.

### Standards Selected

We identified four standards from *Caring for our Children* that support breastfeeding in child care settings: (1) facilities should encourage and support breastfeeding; (2) facilities should have a designated place for mothers to breastfeed; (3) solid foods should not be introduced before infants are four months of age, but preferably six months; and (4) infant formula should not be fed to a breastfed infant without parent permission.

### Review and Scoring of State Regulations

We reviewed child care regulations for all 50 states, the District of Columbia, Puerto Rico, the US Virgin Islands, and the Department of Defense (referred to below as “states”). In early 2012, two researchers read all regulations in their entirety and recorded regulations consistent with the four *Caring for our Children* standards. Agreement between the two reviewers was over ninety percent; differences were reconciled by an additional joint review and discussion. While there were no disagreements about coding, there were six cases where one reviewer noted regulations that the other coder had overlooked. Regulations were coded separately for centers and homes. The date of the most recent update was also recorded. To be counted, regulations needed to use clear language, which could be evaluated as a basis for assessing compliance by regulatory agencies. We scored regulations using a three-category coding system: 0 = *Caring for our Children* standard not addressed; 1 = *Caring for our Children* standard partially addressed; 2 = *Caring for our Children* standard fully addressed. For example, Georgia center regulations stated that “Centers shall have a designated area set aside for breastfeeding mothers to breastfeed”, which was scored as a “1”. Mississippi, on the other hand, received a score of “2” for center regulations, which read “Breast-feeding mothers, including employees, shall be provided a sanitary place that is not a toilet stall to breast-feed their child or to express milk. This area shall provide an electrical outlet, comfortable chair, and nearby access to running water”. Louisiana, which does not have breastfeeding regulations for homes, was coded “0”. This coding system has been used in previous regulatory and policy reviews [8, 13].

### Statistical Analysis

We calculated mean regulation score by state for each of the four breastfeeding standards, for centers and homes, as well as a sum of all regulation scores combined. To examine spatial patterns, we mapped regulations for each breastfeeding standard using ArcGIS 10 (ESRI Inc., Redlands, CA). We assessed levels of global spatial autocorrelation, by regulation, by sum of regulation score, and by facility type, using global Moran’s *I* in GeoDa™. Global Moran’s *I* numerically describes the extent to which areas (states in this case) with similar attributes (breastfeeding regulations) tend to cluster throughout a geographic region (the US) [14]. Moran’s *I* values range from −1 (perfect negative spatial autocorrelation, where areas with dissimilar attributes tend to cluster), through 0 (random distribution of area level attributes), to +1 (perfect positive spatial autocorrelation, where areas with similar attributes tend to cluster). The null hypothesis states that there is no discernible spatial pattern in breastfeeding regulations throughout the US as a whole.

However, this global measure may not be sensitive to highly localized clustering or dispersion in breastfeeding regulations—variations which are important to understand, and which may hold implications both for policy and further research—but which have been averaged over using this global approach [15]. Therefore, in addition to the global test statistic, we also calculated complementary local Moran’s *I* values, allowing specific regions of spatial autocorrelation to be identified as ‘clusters’ or ‘outliers’, which might otherwise be overlooked using a global analysis. In short, the local statistic assesses if there are regional clusters of states with similar regulations, even if these do not sum to an overall global correlation. We used this technique to identify states that had high regulations (standard fully addressed) that were surrounded by other states with similarly high regulations (high–high). We also identified states that had low regulations (standard not addressed) that were surrounded by other states with low regulations (low–low), states with low regulations that were surrounded by states with high regulations (low–high), and states with high regulations that were surrounded by states with low regulations (high-low).

## Results

### Overview

We found substantial variation among states. Overall, the majority of states had, on average, less than one regulation meeting *Caring for our Children* standards (Table 1). Out of a possible 2.0 points for each standard, mean scores were 0.35, 0.20, 0.98, and 0.74 for centers, and 0.17, 0.15, 0.79, and 0.58 for homes. Five states had regulations that partially or fully addressed all four standards for centers, including Delaware, Georgia, Michigan, Mississippi, and Texas. For homes, Delaware and Mississippi included regulations consistent, either partially or fully, with the four standards. Twelve states had a regulation that partially or fully met the standard encouraging a general statement of support for breastfeeding for centers and six states for homes. Nine states required a specific place for mothers to breastfeed at centers, and six states for homes. For centers, 41 states partially or fully regulated the introduction of solid foods, and 33 states did so for homes. The standard requiring parent permission to feed infant formula to breastfed infants was addressed in 39 state regulations for centers and 29 state regulations for homes.

**Table 1:** State regulation score for each of four national breastfeeding standards, for child care centers and family child care homes

State	Facility type	Year of last update	Updated post new CFOC standards	Breast-feeding support	Place for mothers to breastfeed	No solids to infants <four months	No formula without permission
AL	Centers	2009	No	0	0	1	1
	Homes	2009	No	0	0	1	1
AK	Centers	2007	No	0	0	0	0
	Homes	2007	No	0	0	0	0
AZ	Centers	2010	Yes	0	1	0	1
	Homes	2004	No	0	0	0	1
AR	Centers	2010	No	0	0	1	1
	Homes	2010	Yes	0	0	1	1
CA	Centers	2005	No	0	1	1	1
	Homes	2009	No	0	0	0	0
CO	Centers	2011	Yes	0	0	1	1
	Homes	2011	Yes	0	1	2	2
CT	Centers	2011	Yes	0	0	1	1
	Homes	2011	Yes	0	0	1	1
DC	Centers	2007	No	0	0	0	0
	Homes	2007	No	0	0	0	0
DE	Centers	2007	No	2	1	1	1
	Homes	2009	No	2	1	1	1
FL	Centers	2010	No	0	0	0	0
	Homes	2010	No	0	0	0	0
GA	Centers	2011	Yes	2	1	2	1
	Homes	2011	Yes	0	0	2	1
HI	Centers	2002	No	0	0	0	0
	Homes	2002	No	0	0	0	0
ID	Centers	2011	Yes	0	0	0	0
	Homes	2011	Yes	0	0	0	0
IL	Centers	2010	No	1	0	2	1
	Homes	2010	Yes	1	0	1	1
IN	Centers	2003	No	2	0	2	1
	Homes	2001	No	0	0	0	0
IA	Centers	2011	Yes	0	0	1	1
	Homes	2009	No	0	0	0	0
KS	Centers	2008	No	0	0	2	0
	Homes	2008	No	0	0	2	0
KY	Centers	2008	No	0	0	0	0
	Homes	2008	No	0	0	0	0
LA	Centers	2003	No	1	0	1	1
	Homes	–	–	–	–	–	–
ME	Centers	2008	No	0	0	0	0
	Homes	2009	No	0	0	0	0
MD	Centers	2011	Yes	0	0	2	2
	Homes	2011	Yes	0	0	0	0
MA	Centers	2010	Yes	0	0	1	1
	Homes	2010	Yes	0	0	1	1
MI	Centers	2008	No	2	1	1	1
	Homes	2009	No	0	0	0	0
MN	Centers	2010	No	0	0	1	1
	Homes	2007	No	0	0	0	0
MS	Centers	2009	No	2	2	1	1
	Homes	2009	No	2	2	1	1
MO	Centers	2011	Yes	0	0	1	1
	Homes	2011	Yes	0	0	1	1
MT	Centers	2006	No	0	0	1	1
	Homes	2006	No	0	0	1	1
NE	Centers	1998	No	0	0	1	1
	Homes	1998	No	0	0	1	1
NV	Centers	2007	No	0	0	2	1
	Homes	2007	No	0	0	2	1
NH	Centers	2008	No	0	0	2	1
	Homes	2008	No	0	0	2	1
NJ	Centers	2009	No	0	0	2	1
	Homes	2009	No	0	0	2	1
NM	Centers	2010	No	0	0	0	0
	Homes	2010	No	0	0	0	0
NY	Centers	2005	No	2	0	1	1
	Homes	2005	No	2	0	1	1
NC	Centers	2010	Yes	0	2	1	1
	Homes	2010	Yes	0	2	1	1
ND	Centers	2011	Yes	0	0	2	1
	Homes	2011	Yes	0	0	2	1
OH	Centers	2010	Yes	1	0	1	1
	Homes	2011	Yes	1	0	1	1
OK	Centers	2010	No	0	0	1	1
	Homes	2010	Yes	0	0	0	0
OR	Centers	2010	Yes	0	0	2	1
	Homes	2010	Yes	0	0	1	0
PA	Centers	2009	No	0	0	2	1
	Homes	2009	No	0	0	2	1
RI	Centers	1993	No	0	0	1	1
	Homes	2007	No	0	0	1	1
SC	Centers	2005	No	0	0	1	1
	Homes	2005	No	0	0	1	1
SD	Centers	2004	No	0	0	0	0
	Homes	2004	No	0	0	0	0
TN	Centers	2009	No	2	0	1	0
	Homes	2009	No	0	0	1	0
TX	Centers	2010	Yes	1	1	1	1
	Homes	2010	Yes	0	1	1	1
UT	Centers	2009	No	0	0	0	0
	Homes	2011	Yes	0	0	0	0
VT	Centers	2001	No	0	1	2	0
	Homes	2001	No	0	1	2	0
VA	Centers	2008	No	1	0	1	1
	Homes	2011	Yes	1	0	1	1
WA	Centers	2010	Yes	0	0	1	1
	Homes	2010	Yes	0	0	1	1
WV	Centers	2007	No	0	0	1	1
	Homes	2009	No	0	0	1	1
WI	Centers	2009	No	0	0	1	1
	Homes	2009	No	0	0	1	1
WY	Centers	2008	No	0	0	1	1
	Homes	2008	No	0	0	1	1
PR	Centers	2002	No	0	0	0	0
	Homes	2002	No	0	0	0	0
USVI	Centers	2009	No	0	0	1	1
	Homes	2009	No	0	0	1	1
DOD	Centers	1996	No	0	0	0	0
	Homes	1996	No	0	0	0	0

0 = standard not addressed; 1 = standard partially addressed; or 2 = standard fully addressed

*PR* Puerto Rico, *USVI* United States Virgin Islands, *DOD* Department of Defense

### Spatial Clustering

**Fig. 1:**
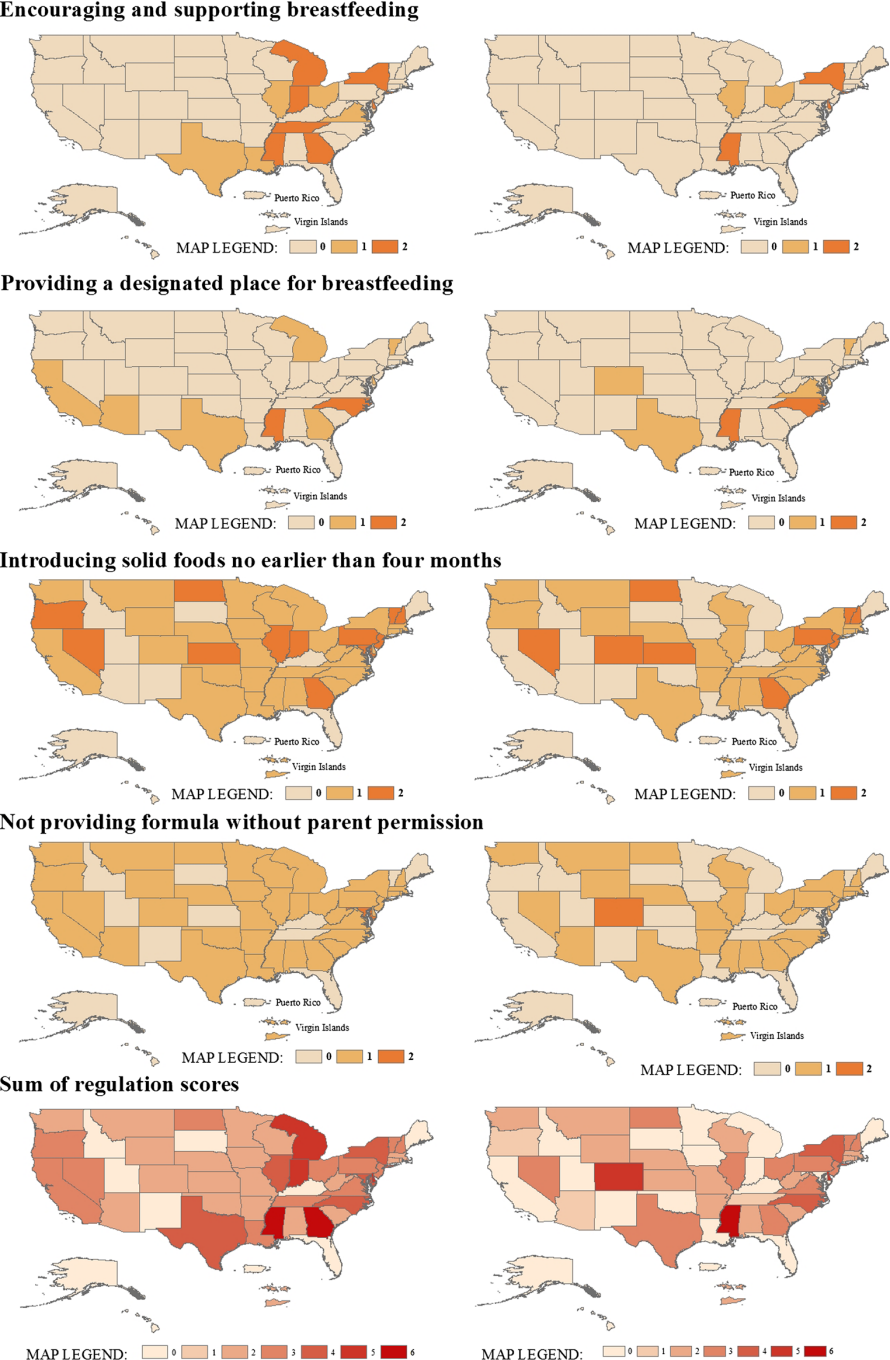
Spatial patterning of regulations for child care centers (*left*) and family child care homes (*right*)

Figure 1 provides a geographic depiction of regulation score by state for each of the four breastfeeding standards, as well as a sum of regulation scores for all standards combined, for centers and for homes. For centers, Georgia and Mississippi had the highest total scores when scores for all four standards were combined. For homes, Colorado and Mississippi had the highest total scores. When we evaluated spatial patterning statistically, Global Moran’s *I* testing revealed no significant spatial autocorrelation for any of the breastfeeding standards for either centers or homes (Table 2). While global Moran’s *I* coefficients ranged from −0.15 (slight negative autocorrelation) to 0.10 (slight positive autocorrelation), none of the tests reached statistical significance for the individual standards or for the sum of the standards combined (Table 2). While the global Moran’s *I* test was not statistically significant, it may not have been sensitive enough to detect the limited number of clusters observed in this study.

**Table 2:** Levels of global spatial autocorrelation (Moran’s *I*) in state breastfeeding regulation score for child care centers and family child care homes

Regulation	Child care centers		Family child care homes	
	Moran’s *I*	*z*-score	Moran’s *I*	*z*-score
Support for breastfeeding	0.100	1.287	−0.092	−0.753
Designated place to breastfeed	−0.060	−0.431	−0.051	−0.298
No solid foods before four months	−0.084	−0.644	−0.032	−0.130
No formula without parent permission	−0.150	−1.342	−0.115	−0.973
Sum of regulation scores	0.040	0.637	−0.041	−0.165

**Fig. 2:**
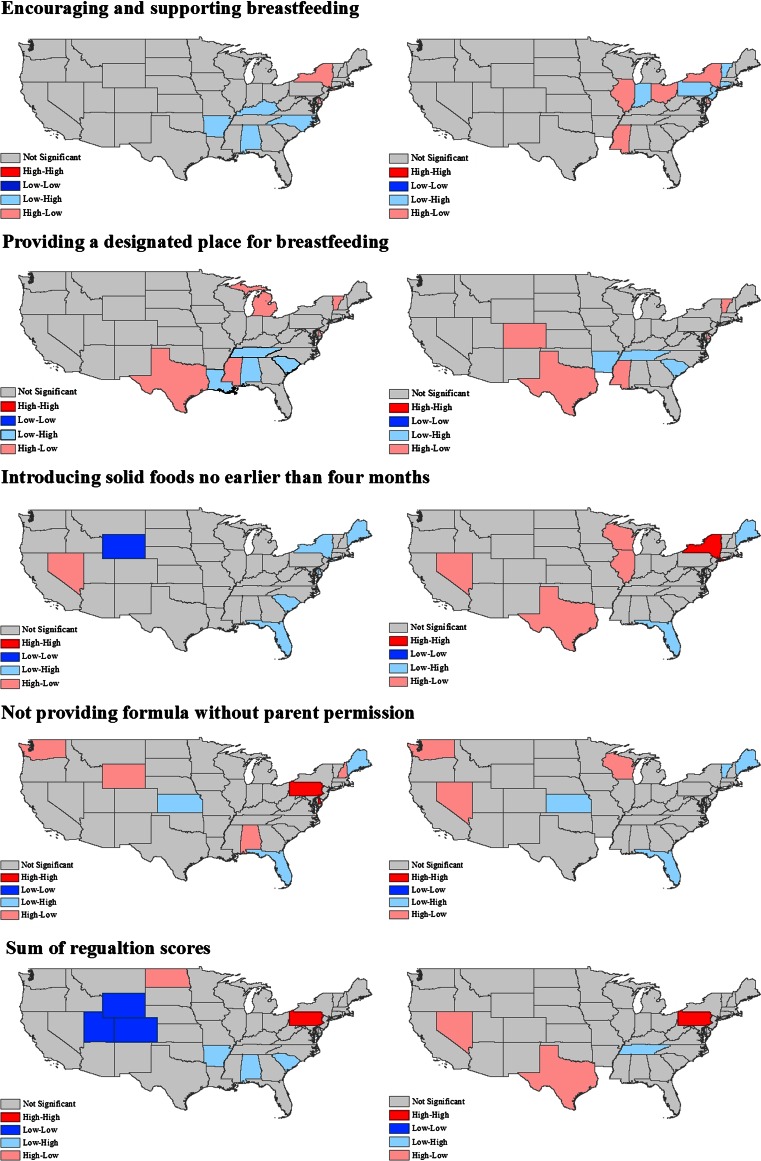
Localized clustering of regulation scores for child care centers (*left*) and family child care homes (*right*)

Conversely, local Moran’s *I* statistics showed significant positive and negative local spatial autocorrelation across all regulations in both settings, contrary to the lack of significant autocorrelation observed using the global measure (Fig. 2). Overall, cases of negative spatial autocorrelation—‘outliers’, states with high standards surrounded by those with low standards (high–low), and vice versa (low–high)—were more prevalent than those of positive autocorrelation (‘clusters’, high–high or low–low). Consistently, states in the southeast tended to be outliers (Florida in particular), yielding lower scores than surrounding states. For the designated place standard in particular, Texas consistently out-performed its neighboring states in both child care settings.

There were only isolated cases of positive spatial autocorrelation (high–high or low–low) observed throughout the US, and none for encouraging and supporting breastfeeding and designated place regulations. Two states with high regulations alongside their neighbors were found in the northeast (New York and Pennsylvania), but for different standards and in different child care settings. Wyoming was the only state identified as belonging to a significant cluster of states with consistently low regulations for the introduction of solid food standard. When we considered the sum of the regulation scores (all standards combined), Pennsylvania was the only state with a high score and high-scoring neighbors (high–high) for both centers and homes (Fig. 2). There was one cluster of states with low scores (low–low), which included Colorado, Utah, and Wyoming for centers only. We did not observe any significant cluster of low scores (low–low) for the sum of regulations for homes.

## Discussion

This review of breastfeeding regulations revealed variation among states. Overall, the mean score for each of the four standards was <1.0 for both centers and homes, below the possible score of 2.0 if the standard was met fully, meaning most states did not have a regulation or if they did, it only partially met the standard. The majority of states had regulations addressing the standard prohibiting the introduction of solid foods to infants <four months of age, as well as regulations requiring parental permission to give formula to breastfed infants. Regulations supporting breastfeeding in general or requiring a designated place for mothers to breastfeed their infants at the child care facility were less common.

Although previous studies have compared child care regulations to national standards [6–10], this is the first to examine the spatial patterning among US states, allowing for a nationwide overview of state level breastfeeding regulations for the first time. We identified a number of states that belonged to spatial clusters of high regulations (New York, Pennsylvania), clusters of low regulations (Colorado, Utah, Wyoming), and others that constituted spatial outliers, such as Texas, which had stronger regulations than its surrounding states. This information bears relevance to both future research and breastfeeding policy, an important aspect of maternal and child health. This analysis should serve to generate hypotheses and guide future, perhaps qualitative research, seeking to derive explanations for these patterns. For instance, where a state with a strong regulation forms part of a cluster, surrounded by other states with strong regulations, researchers and policymakers could seek to understand the mechanisms through which these regulations were proposed and adopted. This information could be applied elsewhere, perhaps in regions where one state outperforms its neighboring states. In addition, as previously stated, geographic analyses can help identify areas of the country with relatively weak breastfeeding policies and target them for intervention. This review may also serve as a baseline study, from which to assess changes in state level breastfeeding policies through time in future research. From a policy perspective, national efforts to promote breastfeeding may also benefit from this identification of breastfeeding regulation clusters and outliers. For example, encouragement of stronger regulatory uptake could be focused toward clusters of states with low levels of breastfeeding regulation, potentially yielding important health benefits for infants in child care in these regions. Consequently, this study builds on and extends previous research on state-level breastfeeding policy and points to several targets for improving breastfeeding policy and therefore improving maternal and child health.

We identified a few states that belonged to spatial clusters of high regulations (such as New York and especially Pennsylvania), while others were associated with clusters of low regulations (for example, Colorado, Utah, and Wyoming). Other states constituted spatial outliers, such as Texas, which had stronger regulations relative to its surrounding states. Identifying these specific clusters of regulations is of policy importance. Targeting these areas for improvement may have important health implications for infants in child care if breastfeeding is more supported through stronger regulation.

Previous research has documented variation in breastfeeding laws among states. The Centers for Disease Control and Prevention (CDC) Breastfeeding Report Card [16] noted that six of the 50 US states (Arizona, California, Delaware, Mississippi, North Carolina, and Vermont) had center breastfeeding regulations ranked as “optimal”, which was similar to our findings. We found five states with regulations that addressed all four standards for centers—at least partially— in our review. Those states included Delaware, Georgia, Michigan, Mississippi, and Texas. For homes, two states (Delaware and Mississippi) included regulations consistent with all four standards. The CDC Report Card included one standard for evaluating state regulations, which was to “encourage and support breastfeeding and feeding of breast milk by making arrangements for mothers to feed their children comfortably on-site”. Thus, our findings are unique in that we assessed four standards supporting breastfeeding in child care.

One study found that infants living in states without breastfeeding promotion legislation (not necessarily specific to child care) had 63 % higher odds of not being breastfed after birth and 45 % higher odds of not being breastfed for at least six months, compared to states with multiple laws [17]. Another study examining organizational policies on worksite lactation support found significant correlation between state laws and rates of exclusive breastfeeding [18]. However, they did not consider regulations targeting child care facilities, where mandated support for breastfeeding mothers may be critical. A third study examined five laws supporting breastfeeding and found that states in the northeast had the most and states in the midwest had the fewest [11]. We observed that states in the northeast and southeast were more likely to have regulations supporting breastfeeding in general, but not for the other standards.

Few studies have prospectively evaluated the effects of breastfeeding laws. One cross-sectional study found that breastfeeding initiation rates were higher in states requiring break time and private space for employees [19]. Laws supporting breastfeeding appeared to impact Hispanic and black women, and women with lower educational levels more than other women. The authors suggest that breastfeeding laws may help reduce racial, ethnic, and socio-economic disparities in breastfeeding [19]. Although previous studies have compared existing child care regulations to national standards [6–10], this is the first study to examine spatial patterns among states, which is a meaningful contribution to the literature.

In this regulatory review, we also identified a few states with regulations that may negatively impact breastfeeding, as some states require child care providers to use universal precautions when handling human milk. For example, homes in Utah must wear waterproof gloves when handling human milk, sanitize any surfaces exposed to human milk, and dispose of human milk in leak-proof plastic bags. Centers in Iowa consider human milk a “bodily excrement”, and thus, providers must use universal precautions. In contrast, Mississippi, Ohio, Texas, and West Virginia (for centers only) state explicitly that universal precautions are not required when handling human milk. Mississippi regulations, for example, require human milk to be handled and stored in accordance to AAP [5] and Centers for Disease Control and Prevention (CDC) [20] guidelines, which state specifically that universal precautions are not necessary.

The presence of regulations supporting breastfeeding is not necessarily related to rates of breastfeeding within the state. In our study, we found that five states had regulations that partially or fully addressed all four breastfeeding standards for centers, including Delaware, Georgia, Michigan, Mississippi, and Texas. For homes, Delaware and Mississippi included regulations consistent, either partially or fully, with the four standards. However, based on the 2012 CDC Report Card (the year of our regulatory review), rates of breastfeeding at six months were highest in Oregon, Utah, Vermont, New Hampshire, and Idaho [17]—five states with relatively few regulations in our policy review. Thus, while this review assesses the presence of regulations, this may not reflect actual practice. Future studies could explore the association between the quality of regulations and practices within child care settings.

At any given time, some states are updating their regulations. Therefore, this research reflects regulations at the time of the review in early 2012. Additionally, state regulations were assessed to see if they had been updated since the revised *Caring for our Children* standards were released in mid-2011. Since regulatory change takes time, states that made recent improvements may not have considered the revised *Caring for our Children* standards before they began their updates. Thus, this review could serve as a baseline to evaluate changes states make in response to the standards. Researchers can help document and evaluate these efforts, but further research is also required to better understand the local patterns of spatial correlation identified in this work.
